# 709 Reconfiguration: Extracorporeal blood purification of a burn patient on ECMO

**DOI:** 10.1093/jbcr/irac012.263

**Published:** 2022-03-23

**Authors:** Anthony P Basel, Jason Thomas, Alicia M Williams, Steven Stoffel, Robert Walter, Phillip Mason, Garrett W Britton, Leopoldo C Cancio

**Affiliations:** United States Army Institute of Surgical Research, Fort Sam Houston, Texas; Brooke Army Medical Center, San Antonio, Texas; United States Army Institute of Surgical Research, Fort Sam Houston, Texas; Brooke Army Medical Center, Ft. Sam Houston, Texas; Brooke Army Medical Center, San Antonio, Texas; Brooke Army Medical Center, Fort Sam Houston, Texas; US Army Institute of Surgical Research, JBSA Fort Sam Houston, Texas; US Army Institute of Surgical Research, JBSA Fort Sam Houston, Texas

## Abstract

**Introduction:**

Patients who require extracorporeal membrane oxygenation (ECMO) have a very high mortality if they develop septic shock. Extracorporeal blood purification has been studied as an adjunct to antimicrobials but has yielded mixed or even disappointing results. The Seraph-100 Microbind Affinity Blood Filter (ExThera Medical Corporation, Martinez, CA) is currently undergoing clinical trials. The filter consists of polyethylene beads, coated in heparin sulfate, that irreversibly binds bacteria, fungi, viruses, and toxins. Seraph-100 therapy is traditionally delivered through conventional hemodialysis or continuous renal replacement therapy (CRRT), with the filter being placed in-line with these circuits. We present a case of a burn patient on veno-venous (VV) ECMO in septic shock, who was treated with a Seraph filter by connecting it directly to the ECMO circuit.

**Methods:**

We present a case.

**Results:**

A 34-year-old male presented with 56% thermal burns and grade 1 inhalation injury from a fuel tank explosion. He underwent a large-volume resuscitation for burn shock with lactated Ringer’s and albumin, receiving 18,152 mL (163 mL/kg) in the first 24 hours. He was placed on CRRT for acute kidney injury and underwent escharotomies of the hands and legs. On day 4, he developed bacteremia, septic shock and progressed to acute respiratory distress syndrome requiring VV ECMO. Extracorporeal blood purification was started via the Seraph-100 filter. Due to limitations of blood flow rates on CRRT, the Seraph-100 filter was added directly into the ECMO circuit. Inflow tubing was connected to an existing port on the oxygenator (Fig 1) and returned to the venous drainage by cutting a new port into the drainage tubing (Fig 2). The filter itself did not require any special configuration or orientation (Fig 3). This configuration allowed for pressures generated by the ECMO circuit to drive blood flow through the Seraph-100 filter (Fig 4). After 6 hours of treatment, vasopressor requirements drastically decreased.

**Conclusions:**

Complications related to the Seraph-100 filter are rare but may include catheter thrombosis. This is typically due to the type of catheter used and/or the blood-flow rate through the filter rather than the filter itself. This issue was avoided with the ECMO configuration. Similarly, clinicians can avoid transient hypotension, blood loss from a clotted circuit, catheter-site bleeding, and other complications frequently associated with a renal replacement circuit.

